# Early results from a multi-component French public-private partnership initiative to improve participation in clinical research – CeNGEPS: a prospective before-after study

**DOI:** 10.1186/s12874-015-0044-8

**Published:** 2015-08-19

**Authors:** Régis Bordet, Marie Lang, Christelle Dieu, Nathalie Billon, Jean-Pierre Duffet

**Affiliations:** Centre National de Gestion des Essais de Produits de Santé, 3 quai des Célestins, BP2251, 69229 Lyon, Cedex 02 France

**Keywords:** Clinical trial organisation, Patient recruitment, Networks of excellence

## Abstract

**Background:**

A public-private (51/49 %) partnership was initiated in 2007 in France to improve the attractiveness of French sites in industry-sponsored international clinical trials. This initiative developed and implemented a combination of structuring actions and support actions. Here we report the assessment of the impact after 6 years on participation of French study sites in industry-sponsored clinical trials.

**Methods:**

We performed a prospective before-after study of clinical research activities in French public hospitals to assess the impact of actions developed and implemented by CeNGEPS. The programme involved a combination of structuring actions (establishment of sites of excellence, national networks and dedicated clinical research assistants (CRAs)), support actions (tools, templates and training) and competitive budget allocation for sites or networks based on performance. The impact was assessed using the following performance criteria: 1) reduction of the delay to contract signature to ≤ 60 days for 80 % of the trial sites; 2) inclusion of ≥80 % of the planned number of patients by at least 80 % of trial sites; 3) closure of <15 % of trials sites without patients enrolled.

**Results:**

In 2013, the median delay to contract signature was: 55 days, compared with 76 days in 2008 (27.6 % reduction), 50.5 % of all sites and 58 % of sites with a dedicated CRA included ≥80 % of the planned number of patients compared with 44.8 % in 2008 (12.7 % increase) and 21.3 % of all sites and 9 % of sites with a dedicated CRA closed with no patients included, compared with 26.4 % in 2008 (19.3 and 65.9 %, respectively).

**Conclusions:**

These results provide evidence that it is possible to improve a country’s attractiveness for industry-sponsored clinical research. The two main actions, i.e. establishing sites of excellence throughout the country with well-trained, dedicated staff and establishing a national network of clinical investigators, could be adapted to other countries in Western Europe to improve Europe’s attractiveness to industry-funded trials.

## Backgound

At the turn of the 21st century in France the clinical research community and public authorities witnessed the decline of France’s role in industry-sponsored international clinical trials [1, 2]. This led to the recognition of the need to improve France’s performance in this area [3].

France has always played a role in the advancement of medical knowledge and innovation, e.g. the discovery of the stethoscope by René Laennec, the development of the principles of experimental medicine by Claude Bernard and the role of Louis Pasteur in the discovery of ‘microbes’. Europe, in general, and France, have become less important for industry-sponsored international clinical trials [1, 2, 4]. France was ranked 3rd among European countries for clinical trials, proportional to the size of its population and its market for pharmaceuticals in the 1990s, although, over time, fewer phase II and III trials and more post-marketing phase IV were being done. In addition, there was a serious under-representation of French investigators and patients in the mega-trials that become more frequent. The number of clinical trials registered with the French drug and medical devices safety agency (ANSM: Agence Nationale de Sécurité du Médicament et des Produits de Santé) fell nearly 30 % in 7 years, from 1467 in 1998 to 1045 in 2005, while the number of international clinical trials remained stable.

The situation for investigator-initiated trials in France improved significantly, when the Hospital Clinical Research Programme (‘Programme Hospitalier de Recherche Clinique’ – PHRC) was launched in 1993. In 2012, the programme’s budget was 53.5 million euros to fund investigator-initiated clinical studies. The initiative reflects the French government’s commitment to develop academic clinical research and it has largely contributed to its progress. However, it did not have any impact on the development of industry-sponsored international clinical research.

In 2001, the European Union Clinical Trials Directive (EUCTD) was adopted with the aim of harmonising and simplifying clinical trial requirements across the European Union, and thereby sustaining European innovation and competitiveness [5]. However, it is generally agreed that it failed and instead, undermined Europe’s position in clinical research by raising legal barriers, and increasing bureaucracy, work load and costs [6]. The rates of Clinical Trial Applications (CTAs) has declined by 1.9, 2.3, 3.0 and 5.3 % on average every year in the Netherlands, Germany, France and the UK, respectively, since 2001 [7]. Southern European countries, such as Spain and Italy seemed to have benefited from the EUCTD with an average annual increase of CTAs of 2.5 and 5.0 %, respectively.

In 2006, a survey by the LEEM, a French organisation representing 270 pharmaceutical companies, responsible for 98 % of pharmaceutical sales in France, concluded that there was a negative perception of the productivity of clinical research in France compared with other countries [8]. High cost, coupled with slow patient recruitment rates and the high number of sites that closed without including patients all contributed to this poor perception. In the setting of globalisation, these factors are not in favour of the implementation of clinical research in France when countries such as India and China and those in Eastern Europe can offer many advantages, particularly in terms of patient recruitment.

The National Centre for the Management of Clinical Trials of Healthcare Products (in French: ‘Centre National de Gestion des Essais de Produits de Santé’ – CeNGEPS), a public-private structure, was established in March 2007 to facilitate the coordination and management of industry-sponsored clinical trials in France. Here we report the activities undertaken and the evaluation of its impact on industry-sponsored clinical research in France after 5 years.

## Methods

### Description of the CeNGEPS initiative

Using the British model of the Pharmaceutical Industry Competiveness Task Force (PICTF) created by Prime Minister Tony Blair, France set up a Strategic Advisory Committee for the Healthcare Industry (in French: ‘Conseil Stratégique des Industries de Santé’ – CSIS) [9, 10]. This committee was composed of the highest political authorities and representatives of the healthcare industries. Their aim was to develop recommendations for improving and maintaining the attractiveness of France for industry-sponsored international clinical trials, in the light of the major economic and political issues at stake; 250 000 to 300 000 jobs in France and a profit of over 7 billion euros [11].

This committee recommended the establishment of CeNGEPS to be at the centre of interactions between the different actors in industry-sponsored clinical trials in France: the healthcare organisations; the investigators; the industrial sponsors; and the patients and healthy volunteers. CeNGEPS is a public-private partnership, with 51 % public associated-members and 49 % private associated-members. Its political and strategic policies are decided by a general assembly comprising 24 members representing its associated-members. A Scientific and Technical Advisory Board organise the calls for proposals, are present the selected proposals to the general assembly for approval. All proposals are assessed by two rapporteurs, one academic and one industrial.

Only 5 % of its annual budget from the government (10 million euros), financed by a tax on the turnover of French pharmaceutical industries, is used for operational costs. The rest of the budget is used for the contact points, one in each of the seven hospital clinical research inter-regions and the annual call for proposals which provide funds for staff, particularly clinical research technicians (CeNGEPS-CRTs) who assure the management and coordination of industry-sponsored clinical trials. In 2012, 280 full-time-equivalents (FTE) posts (including 190 FTEs-CRTs) were funded.

Based on bi-annual surveys by the LEEM and its own survey after its creation, CeNGEPS identified six main weaknesses that orientated and guided their program of structuring and support actions (Tables 1 and 2) [12–17]. The structuring actions involve two annual calls for projects to provide financial support for the development of sites of excellence in clinical research and national clinical investigator networks (Table 2). The support actions involve the development of tools to support the sites of excellence and networks. In particular, one of the support actions was the development of a website to increase the public awareness about clinical research and its importance (Table 2). In addition, CeNGEPS introduced a system of competitive budget allocation for sites or networks based on performance.

**Table 1:** Identified weaknesses of the French clinical research environment and remedial actions undertaken to make it more attractive and competitive [12–17]

Identified weaknesses	Remedial actions
Disinterest in industry-sponsored clinical research	Creation of a single-point of contact for industries by funding the following posts:
• No clearly-identified referent	• Inter-regional contact point for industry
	• Industrial referent (and administrative assistant) in the DCRI in each teaching hospital
	• Positive image of French clinical research by the professionalism and dynamic structures with a culture of results, particularly in terms of patient recruitment delays and objectives
Fragmented clinical research resources between research institutes and hospitals/clinics	• No additional structure but annual call for proposals for CRT posts to strengthen the existing inter-regional structures
	• Development of tools for feasibility surveys
	• Funding of CRTs in non-teaching hospitals to increase capacity for patient recruitment
Confusion about the meaning of ‘network’	Annual funding call for ‘support for clinical investigation network’:
• In the hospital setting, ‘network’ is often associated with care or healthcare networks	• Development of a thematic national clinical investigation networks for international collaborations
	• Definition of missions and clear added value
	• Development of standards and performance criteria
No common information system	Development of SIGREC, an information system dedicated to the administrative management and follow-up of clinical trials (industry-sponsored and investigator initiated)
	• Faster implementation of clinical trials
	• One-stop information source for all sites – assess availability for new trials
	• Long-term aim – to reduce paperwork and provide statistics on number of studies/investigator etc.
Long delay before clinical trial implementation	• Development of model contracts and financial
	• Appendices (in French and English) ➔ save time
	• In 2014, possibility to set up contract via SIGREC (paperless environment)
Isolated investigators	• Almost 500 qualified clinical research staff have been hired and trained via three calls for proposals
• Rarely associated with specific, qualified CRTs or other staff	• These staff are dedicated to industry-sponsored clinical research
	• The professionalism of the teams and the improved organisations of the sites have contributed to a more attractive and competitive clinical research environment in France

*DCRI* Department of Clinical Research and Innovation, *CRT* clinical research technician

**Table 2:** Summary of the structuring and support actions undertaken by CeNGEPS

Structuring actions	1. Aid to establish sites of excellence recognised by the hospital clinical research inter-regions:
	*Annual call for proposals for the seven clinical research inter-regions to fund dedicated CRTs and other qualified staff to help coordinate studies and patient inclusion (pre-screening and screening)*
	2. Aid to establish national networks:
	*Annual call for proposals for the 23 national clinical investigation networks for project managers and network animators and the implementation of a database*
Support actions	i. Collaborative development of models for contracts, financial annexes and national harmonisation of documents between hospitals/clinics and industrial sponsors in conjunction with a unique industrial office and contact point in the hospitals/clinics
	ii. Automatic tool to assess the patient database to prevent study sites closing with no patients included (estimated to cost sponsors about €12 000/centre) through feasibility surveys
	iii. Aid for the development of potential investigators and access to patients in non-teaching hospitals/clinics
	iv. National programmes for professionalization and training in clinical research to guarantee quality
	v. Increasing public awareness about clinical research and its importance in society via a website (www.notre-recherche-clinique.fr) which went live in April 2010; 329 760 visits and 1 346 883 pages seen up to August 2013

### Assessment of the impact of CeNGEPS

CeNGEPS developed and implemented follow-up tools in April 2007 to evaluate the impact of the combined actions. Using these tools, reliable data have been collected since 2008 to measure the impact. One of these tools, implemented on the CeNGEPS extranet, enables real-time follow-up and analyses of the CRTs’ activity, as well as financial surveillance of attributed funds. The second tool, not on the CeNGEPS extranet, enables information on the overall activity of industry-sponsored clinical research in the inter-regions to be collected using an Excel spreadsheet; the inter-region point of contact is responsible for collecting the data from the various sites in their region.

The sites are classified as ‘new’ or ‘closed’. New sites are those that have signed a contract with an industrial sponsor during the year being assessed and closed sites are those that are closed to recruitment and have invoiced the sponsor in the year being assessed. When all the files are received, the data are analysed to produce annual activity reports.

### Definition of performance criteria

To measure the impact of these actions, other than counting how many patients were included in industry-sponsored clinical trials, with a panel of pharmaceutical companies representatives, three international gold-standard performance criteria for the inter-regional centres were defined based on their experience of criteria used in their companies to judge the efficiency of a clinical investigation site [12, 18].

- delay to contract signature: 60 days for 80 % of the contracts
- 80 % inclusion rate in 80 % of the sites including patients
- <15 % of sites closing with no patients included

The inter-regional funding has an integrated bonus/penalty scheme to reward or penalise the inter-regions that achieve these criteria or not, by increasing or reducing their annual budget.

## Results

### Impact on performance criteria

After 6 years of effective activity, the median delay to contract signature was reduced from 76 days in 2008 to 55 days in 2013 (27.6 %). In 2013, 53.2 % of sites reached the objective of having 80 % of the contracts signed within 60 days, compared with 39.0 % in 2008 (36.4 % increase).

There was a 12.7 % increase in the number of sites that included >80 % of the planned number of subjects. The mean number of patients included by centre was 1.8 % higher in 2013 than in 2008. There was a 19.3 % reduction in the number of sites that closed without having included any patients. There was a 5.8 % increase in the number of sites that closed and billed their industrial sponsor in 2012 compared with 2008 (Table 3).

**Table 3:** After 6 years of CeNGEPS activities: overall activity for industry-sponsored clinical research in the French inter-regions

Outcome	2008	2013	Percentage change
Sites (closed and invoiced)	2192	2057	−6.1
Median inclusion rate (%)	40	57.1	+42.3
Number of included patients	11 837^a^	11 740	−0.8
Mean number of patients included/centre	5.6^a^	5.7	+1.8
Inclusion rate in sites (closed and invoiced) (%)	63^a^	75.6	+20
% of sites with no patients included	26.4	21.3	−19.3
% sites included >80 %	44.8	50.5	+12.7
Median delay to contract signature (days)	76	55	−27.6
% reaching objective of 80 % contracts signed ≤60 days	39.0	53.2	+36.4

^a^2009 data used because 2008 data were incomplete

### Assessment of the impact of CeNGEPS-funded clinical research technicians

In 2013, 58 % of sites with a CeNGEPS-CRT included >80 % of the number of patients targeted compared with 47.0 % of sites without a CeNGEPS-CRT. Only 9.0 % of sites with a CeNGEPS-CRT closed with no patients included, compared with 26.0 % of those without a CeNGEPS-CRT. The median inclusion rate was higher in the sites with a CeNGEPS-CRT: 80 vs 50 %.

Data from the CeNGEPS extranet showed that, in sites with a CeNGEPS-CRT, there were 873 trials closed to recruitment in 2013 compared with 670 in 2011 (+30 %). There was an increase in the median recruitment rate, from 71.4 to 89.0 %, and the percentage of sites that recruited >80 % of their target slightly increased from 58.7 to 58.9 %. There were fewer sites closed with no patients recruited (17.2 vs. 9 %; −47.7 %).

In 2013, there were 267 on-going, industry-sponsored trials in the 22 CeNGEPS networks that included 2 608 patients (1 % more than the planned 2 581 patients). Three networks included ≥100 % of the planned number of patients in all their trials. Eight networks achieved the target of ≥80 % of their sites with ≥ 50 % of the planned number of patients included; 72.6 % of the sites in the networks reported inclusion of ≥50 % of the planned number of patients. The percentage of sites that closed having included no patients was 12.4 %; 5 of the networks had no sites closed with no patients recruited. In the 2013 call for proposals, 16 of the 23 networks received bonus funding (i.e. >100 % of planned funding) because they had satisfied ≥1 of the quantitative or qualitative performance criteria defined by CeNGEPS. There was a move to out-source industry-sponsored clinical research to clinical research organisations (CROs); in 2013 46 % of the new sites outsourced by sponsors. Among the sites that outsourced the mean delay to signature was 60 days (50 % of the sites had a delay ≤60 days) compared with 51 days (56 % ≤60 days) for sites who did not outsource to a CRO.

## Discussion

The analyses presented clearly show that there has been a positive impact on industry-sponsored clinical research in France. In addition, for the first time in many years, there was an increase in the number of authorised drug clinical trials to the ANSM: 899 in 2013 compared with 705 in 2012 (decrease from 790 in 2008 to 704 in 2011); about 2/3 of these trials were industry sponsored [19]. The analyses were not stratified by pathology, but cancer accounted for most of the clinical research and the inclusion indicators were high for cancer trials. A recent meta-analysis showed that cancer trials were associated with successful recruitment; other factors included having a dedicated trial manager, being a drug trial and testing an intervention only available in a trial setting [20]. There is always a lag between the implementation of actions and the impact becoming visible. For example, the number of sites that closed with no inclusions significantly decreased for the first time in 2012 to 20 % and confirmed its trend in 2013, after having stagnated at about 26 % from 2008. In 2013, the implementation of a new tripartite contract template was probably responsible for the 4-day longer delay to contract signature, compared with the delay in 2012.

Delays in time to contract signature can be explained because many actors are involved and discussions between the trial sponsor and the investigators and between the trial sponsor and the hospital are necessary to reach agreement on budget and administrative responsibilities for signature. CeNGEPS was not able to influence all factors in this complex process, but by providing dedicated personnel, we were able to improve the relationship between the trial sponsor and the investigators for initiating trials and for the follow-up of the process, including faster budget calculation and simpler administrative circuits.

Maintaining France as an attractive country for international industry-sponsored trials will continue to be a challenge, with increasing competition from emerging countries in Asia, South America, and Eastern Europe. In addition, the major transformation of the pharmaceutical industry has an important influence on the French economy; therefore, maintaining the attractiveness of France for clinical research is a major strategic challenge.

By 2020, the French clinical research sites should be self-sufficient. However, many questions remain. For example, how can we assure the continuity of the model and the various actions? How can this model be adapted in other European countries? What can and should be done for clinical research on medical devices and post-marketing studies that currently do not fall within the remit of CeNGEPS? What new tools are needed? How can physicians in private practice be encouraged to participate in clinical research?

The results presented here about this innovative public-private partnership, set up in 2007, show that it is possible to improve the attractiveness of a particular country for industry-sponsored international clinical trials by developing the necessary sites of excellence and networks for clinical research. This success was achieved through funding of hospital and networks, mainly to recruit personnel dedicated to patient inclusion in clinical trials. Although the specificities of the organisation of teaching and non-teaching hospitals and clinics in France may mean that the actions may not be directly transferred to other countries, the philosophy of the approach could be adapted to form a base for the promotion of industry-sponsored clinical research.

Nevertheless, there are two potential limitations. Firstly, CeNGEPS worked in a global context that could change and favour the attractiveness of a country for clinical trials, with confounding factors such improved medical training for clinical trials, academic-driven patient cohort recruitment, new hospital organization with high-level of medical equipment. Secondly, because CeNGEPS’s annual budget was limited, funding was allocated on the basis of an evaluation; we cannot exclude the possibility of selection bias, since it was impossible to organize a ‘clinical trial’ with random allocation of funding and a comparison of the performance of centres with or without funding. However, we can compare our results with those for the cardiovascular and metabolism fields that were not initially funded by CeNGEPS. These medical fields suffered an important loss of attractiveness until CeNGEPS provided funding for two dedicated networks.

## Conclusions

The CeNGEPS initiative demonstrates that it is possible to improve a European country’s attractiveness for industry-sponsored clinical research with measures. The two main actions, i.e. establishing sites of excellence throughout the country with well-trained, dedicated staff and establishing a national network of clinical investigators, could be adapted to other countries in Western Europe to improve Europe’s attractiveness to industry-funded trials.

## Abbreviations

CeNGEPS: Centre national de Gestion des Essais de Produits de Santé (National Centre for the Management of Clinical Trials of Healthcare Products)
CRT: Clinical research technician
DCRI: Department of Clinical Research and Innovation
PHRC: Programme Hospitalier de Recherche Clinique (Hospital Clinical Research Programme)
EUCTD: European Union Clinical Trials Directive
CTAs: Clinical Trial Applications
